# The Role of Defensins as Pollen and Food Allergens

**DOI:** 10.1007/s11882-023-01080-3

**Published:** 2023-05-13

**Authors:** Valentina Cosi, Gabriele Gadermaier

**Affiliations:** Department of Biosciences and Medical Biology, Paris Lodron University Salzburg, Hellbrunnerstraße 34, 5020 Salzburg, Austria

**Keywords:** Defensin, Pollen food syndrome, Weed pollen allergy, Defensin-related food allergy, Celeriac, IgE

## Abstract

**Purpose of Review:**

Defensin-polyproline–linked proteins are relevant allergens in Asteraceae pollen. Depending on their prevalence and amount in the pollen source, they are potent allergens, as shown for the major mugwort pollen allergen Art v 1. Only a few allergenic defensins have been identified in plant foods, such as peanut and celery. This review provides an overview of structural and immunological features, IgE cross-reactivity, and diagnostic and therapeutic options regarding allergenic defensins.

**Recent Findings:**

We present and critically review the allergenic relevance of pollen and food defensins. The recently identified Api g 7 from celeriac and other allergens potentially involved in *Artemisia* pollen-related food allergies are discussed and related to clinical severity and allergen stability. To specify *Artemisia* pollen-related food allergies, we propose the term “defensin-related food allergies” to account for defensin-polyproline–linked protein-associated food syndromes.

**Summary:**

There is increasing evidence that defensins are the causative molecules in several mugwort pollen-associated food allergies. A small number of studies have shown IgE cross-reactivity of Art v 1 with celeriac, horse chestnut, mango, and sunflower seed defensins, while the underlying allergenic molecule remains unknown in other mugwort pollen-associated food allergies. As these food allergies can cause severe allergic reactions, identification of allergenic food defensins and further clinical studies with larger patient cohorts are required. This will allow molecule-based allergy diagnosis and a better understanding of defensin-related food allergies to raise awareness of potentially severe food allergies due to primary sensitization to *Artemisia* pollen.

## Introduction

Since the identification of the major mugwort pollen Art v 1, several proteins harboring a plant defensin domain are recognized as allergens. Those molecules play an important role in pollen allergy, and recent studies suggest their involvement in food and contact allergy. This review provides a comprehensive overview on allergenic defensins and in particular their potential contribution in various pollen food syndromes.

## Structure and Biological Function of Plant Defensins

**Fig. 1:**
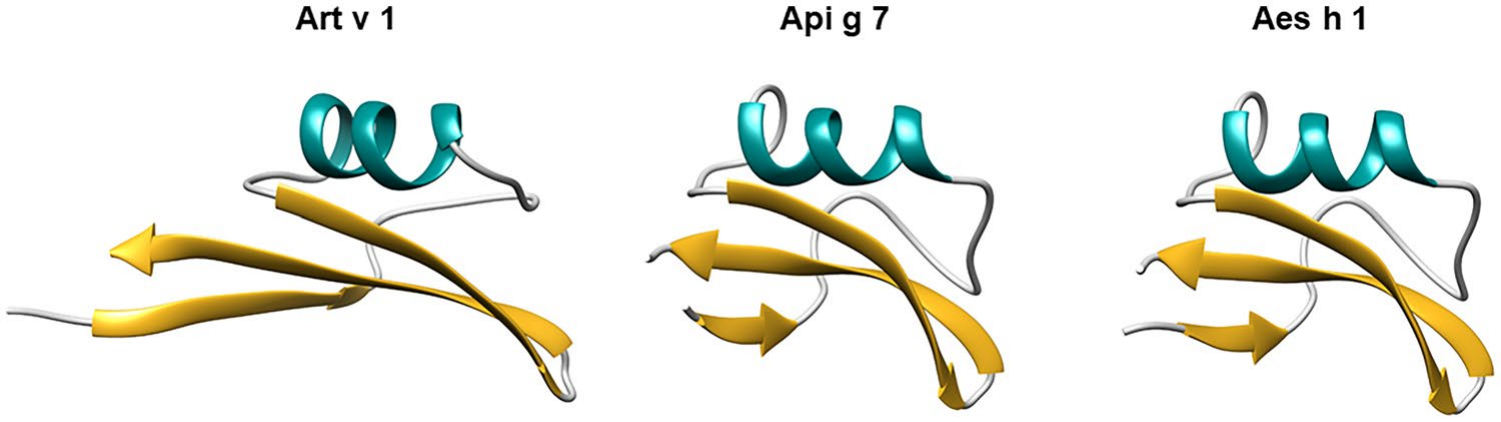
Representation of the defensin-fold originating from pollen, food, and non-edible food. Three-dimensional structures of Art v 1 (PDB 2KPY), model of Api g 7 (generated by SwissModeller using 1BK8 as template) and Aes h 1 (PDB 1BK8). Beta-sheets are indicated in gold, alpha helices in turquoise

Defensins are ancient antimicrobial peptides in plants, insects, invertebrates, and humans. They have a tissue-specific expression pattern, an N-terminal signal, and a highly divergent mature protein sequence. Plant defensins, also termed γ-thionins, are 45–54 amino acid proteins ubiquitously found in the plant kingdom, constituting part of the innate defense system. They typically contain eight conserved cysteine residues and share a βαββ pattern with 3-stranded antiparallel β-sheets and a short α-helix (Fig. 1) [1–3]. The disulfide bond-stabilized αβ-motif confers a compact globular structure and high resistance to extreme pH, temperature, and protease digestion. Most defensins have been isolated from seeds but were also found in leaves, flowers, roots, and stems [4]. More than 2000 plant defensins (IPR008176) and 1000 defensin-like proteins (IPR010851) are listed in the InterPro Classification of protein families (https://www.ebi.ac.uk/interpro). As no clear distinction between defensins and defensin-like proteins is provided, proteins harboring either of the domain will be termed defensins throughout this review.

Plant defensins can inhibit growth of microbes and were thus classified into the pathogenesis-related (PR)-12 protein family. Defensins are predominately expressed in peripheral cell layers, in accordance with their role as first line of defense against plant pathogens. The interaction of positively charged defensins with negatively charged microbial membranes can increase membrane permeability, potentially leading to cell leakage and death [5, 6]. The core motif (GXCX3-9C) formed between the β2 and β3 strands has been proposed to confer antimicrobial activity [7]. Besides their predominant antifungal, and to a lesser extent, anti-bacterial properties, defensins also respond to stress and are involved in plant growth regulation and developmental processes [5, 8, 9]. To enhance pathogen resistance, heterologous defensins were overexpressed in transgenic tomatoes, rice, potatoes, and other plants [5]. However, the fact that defensins can be allergens raise concerns about their introduction into genetically modified foods.

## Allergenic Proteins Harboring a Defensin Domain

**Fig. 2:**
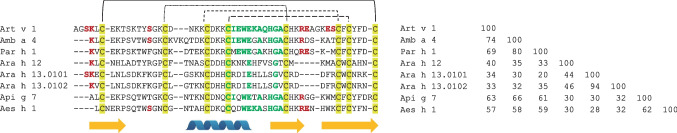
Sequence alignment and identity matrix of defensin domain from pollen and food allergens. Art v 1 B cell epitopes (red) and T cell epitopes (green) and conserved amino acids are indicated, conserved cysteine residues are highlighted in yellow, and disulfide bonds are indicated by bars. Beta-sheets are presented in gold, alpha helices in blue. Sequence identity matrix was generated by Clustal 2.1

Besides the omnipresence of defensins in the plant kingdom, only 20 are officially acknowledged as allergens according to the WHO/IUIS allergen nomenclature sub-committee. Art v 1, the first allergenic representative, was identified as a defensin-polyproline–linked protein (DPLP) in mugwort pollen. Allergens sharing this architecture were later described in ragweed and feverfew pollen. Allergenic defensins without a C-terminal fusion were discovered in few food sources like peanut, celery, and soybean, as well as in horse chestnut (Table 1 and Fig. 2).

**Table 1:** Allergens harboring a defensin domain

**Allergen**	**Latin name**	**Common name**	**Plant family**	**Biochemical description**	**Exposure route**	**MW (kDa)**
Art v 1	*Artemisia vulgaris*	Mugwort	Asteraceae	DPLP, O-glyc	Airway	24–28
Art ab 1	*Artemisia absinthium*	Absinthe wormwood	Asteraceae	DPLP, O-glyc	Airway	24–26
Art an 1	*Artemisia annua*	Sweet wormwood	Asteraceae	DPLP, O-glyc	Airway	24–26
Art an 1	*Artemisia annua*	Sweet wormwood	Asteraceae	DPLP, O-glyc	Airway	28
Art ar 1	*Artemisia argyi*	Silvery wormwood	Asteraceae	DPLP, O-glyc	Airway	28
Art c 1	*Artemisia californica*	California sagebrush	Asteraceae	DPLP, O-glyc	Airway	24–26
Art ca 1	*Artemisia capillaris*	Wormwood	Asteraceae	DPLP, O-glyc	Airway	28
Art f 1	*Artemisia frigida*	Desert sagebrush	Asteraceae	DPLP, O-glyc	Airway	24–26
Art gm 1	*Artemisia gmelinii*	Russian wormwood	Asteraceae	DPLP, O-glyc	Airway	28
Art la 1	*Artemisia lavandulifolia*	Lavender leaved mugwort	Asteraceae	DPLP, O-glyc	Airway	28
Art l 1	*Artemisia ludoviciana*	Silver wormwood	Asteraceae	DPLP, O-glyc	Airway	24–26
Art si 1	*Artemisia sieversiana*	Sieversian wormwood	Asteraceae	DPLP, O-glyc	Airway	28
Art t 1	*Artemisia tridentata*	Common sagebrush	Asteraceae	DPLP, O-glyc	Airway	24–26
Amb a 4	*Ambrosia artemisiifolia*	Short ragweed	Asteraceae	DPLP, O-glyc	Airway	28–30
Par h 1	*Parthenium hysterophorus*	Feverfew	Asteraceae	DPLP, O-glyc.?	Airway	24
Ara h 12	*Arachis hypogaea*	Peanut	Fabaceae	Defensin	Ingestion	8
Ara h 13	*Arachis hypogaea*	Peanut	Fabaceae	Defensin	Ingestion	8
Api g 7	*Apium graveolens*	Celery	Apiaceae	Defensin	Ingestion	16
Gly m 2	*Glycine max*	Soybean	Fabaceae	Defensin	Airway, ingestion?	8
Aes h 1	*Aesculus hippocastanum*	Horse chestnut	Sapindaceae	Defensin	Contact, ingestion?	10

*DPLP* defensin-polyproline–linked protein, *O-glyc.* natural protein is O-glycosylated, *MW* apparent molecular weight of natural protein in denaturing and reducing gel electrophoresis

### Allergenic Defensin-Polyproline–Linked Proteins in Pollen

Allergenic defensins linked with a polyproline-rich region show a typical “head” and extended “tail” structure and were so far exclusively identified in some weed pollen of the Asteraceae family (Table 1). Due to climatic changes, prolonged pollen seasons, augmented antimicrobial, and stress response, the expression of those defensins might increase in plants [10–12]. The N-terminal globular defensin domain is linked to a C-terminal polyproline-rich left-helical structure with O-glycosylations. The flexible proline-rich region can vary in its net charge and might act as an anchor in the plant cell wall [2, 13–16]. In gel electrophoresis, natural DPLPs appear around 24–30 kDa, while the mass spectrometry-derived mass including glycans is around 13–19 kDa. Recombinant non-glycosylated Art v 1 consistently migrates at 19 kDa in gel electrophoresis; its measured and calculated mass is however 10.8 kDa. This unusual migration in gel electrophoresis is likely attributable to the unstructured polyproline-rich region.

#### Art v 1 from Mugwort Pollen and Homologs

Mugwort (*Artemisia vulgaris*) is an allergenic weed of the Asteraceae family flowering in late summer and autumn. *Artemisia* species are relevant allergen sources in Asia, Europe, and Northern America with sensitization frequencies up to 41% among pollen-allergic patients [10, 17]. Art v 1 is the major allergen from mugwort pollen and the best described allergen with a DPLP architecture [15]. It consists of a 55 amino acid defensin domain linked to a 53 amino acid proline-rich region containing several (Ser/Ala)(Pro)2–4 repeats. Natural Art v 1 isolated from pollen is a complex mixture of different isoallergens and glycosylation variants with a measured molecular mass between 12.9 and 16.3 kDa. The cysteine-stabilized defensin-fold confers very high stability to thermal and acidic treatment [18••]. The solution NMR structure of Art v 1 (PDB 2KPY) confirms the disulfide bond pattern of defensins (Fig. 1). While the C-terminus is flexible, the transitional region (Art v 1_57–70_) is partially organized and interacts with the defensin domain [2]. Two novel types of plant-specific hydroxyproline-linked O-glycosylations consisting of a large arabinogalactan and single, but adjacent β-arabinofuranoses were described in the proline-rich region [14]. Art v 1 has a basic net charge in contrast to other DPLPs. To date, highly homologous allergens with 94–99% sequence identity (mostly located in the polyproline region) and varying glycosylation patterns were described in 14 *Artemisia* species [19, 20].

Art v 1 and homologs account for up to 95% IgE reactivity in *Artemisia* pollen-allergic patients [15, 21]. The majority of the conformational IgE epitopes are established on the defensin domain and lost upon disruption of the disulfide bonds [15, 22, 23, 24•]. Using human serum, interacting residues establishing two IgE-binding epitopes were mapped. These epitopes are located on the Art v 1 defensin and transitional domain [2]. A recent study confirms the conformational nature of IgE epitopes [24•]. Interestingly, the binding of patients’ IgE could be blocked with IgG rabbit antibodies raised against the C-terminal region suggesting an additional conformation epitope in the proline-rich region. As part of the C-terminal region interacts with the defensin domain [2], the authors suggest that rabbit antibody binding to the polyproline region could have prevented human IgE binding to its known epitopes due to sterical hindrance. Art v 1 peptides lacking 3-dimensional structure showed no IgE reactivity which together with the novel epitope could aid in the development of a molecular B cell epitope vaccine [24•]. In addition, IgE directed against single arabinosides of Art v 1 is detectable in some patients; however, their biological activity in mediator release assays seem limited [25].

The extensively studied T cell response of Art v 1 revealed, in contrast to other allergens, a single immunodominant T cell epitope Art v 1_25–36_ [26]. In addition, mugwort pollen allergy is restricted by the expression of HLA-DRB1*01 in mugwort pollen-allergic patients [27]. A Jurkat T cell line expressing the human receptor specific for the immunodominant T cell epitope of Art v 1 showed cross-reactivity with allergens from other *Artemisia* species but not for the ragweed and feverfew homolog [18••,^20^, ^28^].

#### Amb a 4 from Ragweed Pollen

Pollen of ragweed (*Ambrosia artemisiifolia*) is an important allergen source in Northern America, Europe, and parts of Asia. Among pollen-allergic patients, sensitization to ragweed can reach up to 54%. The majority of IgE reactivity is however directed against the pectate lyase Amb a 1 [10, 29]. In contrast, moderate allergenicity is attributed to Amb a 4, a DPLP that predominates in glycine and proline residues which contribute to the acidic net charge of the molecule [13]. The glycosylated C-terminal part shows hydroxyproline-linked arabinogalactans, although the level of IgE-binding β-arabinosyl residues is lower compared to Art v 1. Several isoforms of Amb a 4 were determined giving rise to different glycan moieties of around 6 kDa. IgE-binding frequency to Amb a 4 was ~ 20% in ragweed allergic patients but higher in mugwort co-sensitized populations. Among weed-allergic patients, positive IgE reactivity to Amb a 4 was 42% (Austria), 39% (Canada), and 25% (Korea) and typically associated with Art v 1 sensitization [18••]. Forty-two percent of Art v 1 positive patients also reacted to Amb a 4. However, 10% of Art v 1-negative patients showed specific IgE to Amb a 4, indicating some unique epitopes for both allergens.

#### Par h 1 from Feverfew Pollen

Pollen of feverfew (*Parthenium hysterophorus*) was tested skin prick test positive in fall pollinosis patients from the USA and atopic dermatitis patients from India [10]. The DPLP Par h 1 was identified by mass spectrometry of pollen and the sequence obtained by cDNA cloning. The protein shows higher sequence identity with Amb a 4 also presenting an acidic net charge. Among Austrian weed pollen-allergic patients and Indian feverfew reactive individuals, a sensitization prevalence of 60% and 40%, respectively, was demonstrated [30]. In tested weed-allergic patients’ cohorts, IgE reactivity to Par h 1 was 47% (Austria), 42% (Canada), and 29% (Korea) and associated with Art v 1 [18••].

### Allergenic Defensins in Plant Food Sources

In plant food and horse chestnut seeds, a limited number of allergenic defensins has been described (Table 1, Fig. 2). In contrast to the pollen allergens, those representatives only consist of a defensin domain. As those molecules are difficult to extract from the respective natural sources, the number and clinical relevance of allergenic defensins might currently be underestimated.

#### Ara h 12 and Ara h 13 from Peanut

Peanut (*Arachis hypogaea*) allergy is the leading cause of fatal anaphylactic reactions worldwide with a global sensitization prevalence of 1–2% with rising tendencies [31]. Besides the major allergens, three defensins were identified in roasted peanuts [32•]. The natural allergens were purified in one fraction using lipophilic chloroform/methanol extraction and their sequences confirmed by mass spectrometry. As they showed only 43–45% sequence identity, they were termed Ara h 12 and Ara h 13. The peanut defensins with a mass of ~ 5 kDa migrate at 8 kDa under reducing conditions in gel electrophoresis, while they appear as three separate bands under non-reducing conditions. Three of eleven patients’ sera showed a strong IgE reactivity to all peanut defensins and three a weaker and more divergent reaction in non-reducing immunoblots. IgE immunoblots under reducing conditions showed no signals suggesting that disulfide bonds are critical for epitope formation. Peanut defensins demonstrated an antifungal activity by inhibiting the growth of *Cladosporium* and *Alternaria*.

#### Api g 7 from Celeriac

*Apium graveolens* is consumed worldwide as a fresh vegetable but is also frequently included in spice mixtures. It can trigger severe allergic reactions, and thus, declaration is mandatory in the EU. Both stalks and tuber (celeriac) are frequently consumed, yet they differ in their allergen distribution [33]. Api g 7, a novel celeriac defensin, was obtained by cDNA cloning, and its presence was confirmed in celeriac extract [34••]. Api g 7 was recombinantly produced demonstrating a mass of 11 kDa while migrating at 16 kDa in reducing gel electrophoresis. All eight sera from celeriac allergic patients showed IgE reactivity against Api g 7 and also tested positive for Art v 1. Using mediator release assays, the allergenicity of Api g 7 was investigated showing up to 60% of maximal mediator release as compared to 70% induced by Art v 1. Celeriac defensin is highly heat stable as it started unfolding at 74 °C and has a calculated melting temperature of 100 °C. Interestingly, allergy diagnosis using celery extract missed 5/8 patients reactive to Api g 7, suggesting an underrepresentation of the defensin in the extract and/or insufficient extraction methods [33].

#### Gly m 2 from Soybean

Gly m 2 from soybean (*Glycine max*) hull was identified in 1997 and associated with respiratory symptoms in Spanish field workers exposed to the plant. The purified allergen of 8 kDa was recognized by five sera of asthmatic patients. Sequence identification of the allergen is limited to the first 17 amino acids determined by Edman degradation. Gly m 2 shares high sequence similarity with Ara h 12 but was not further investigated [35].

#### Aes h 1 from Horse Chestnut Seed

Horse chestnut, *Aesculus hippocastanum*, is a tree widespread throughout Europe and in parks worldwide. While frequently consumed by horses and deer, the seeds of horse chestnuts are also used in cosmetic/medicinal products and dietary supplements for humans. However, rare but clinically confirmed allergic symptoms to horse chestnut upon handling the seeds were observed (Cosi et al., manuscript in preparation). Upon purification of an IgE-reactive protein migrating at 10 kDa in gel electrophoresis, the protein was identified as *Aesculus hippocastanum* antimicrobial protein 1 (AhAMP1) and officially termed Aes h 1. The 3-dimensional structure of Aes h 1 (PDB 1BK8) demonstrates similarity with the defensin domain of the major mugwort pollen allergen Art v 1 (Fig. 1). The protein has a mass of 5.8 kDa and migrates at 10 kDa in reducing gel electrophoresis. Among subjects who tested positive with horse chestnut seeds in prick-to-prick tests, all reacted to Aes h 1 in ELISA.

### IgE Cross-Reactivity of Defensins in Pollen

Extensive IgE cross-reactivity was shown for highly homologous isoallergens of *Artemisia* species [20, 22]. Homologs from ragweed and feverfew showed a considerable yet patient and geographically tailored cross-inhibition with Art v 1 [18••]. While weed-allergic patients from Canada showed similar reactivity to mugwort, ragweed, and feverfew pollen defensins, Korean patients predominantly reacted only to Art v 1. Although Art v 1 seems to be the most relevant allergen in this protein family, Amb a 4 and Par h 1 bear some unique epitopes that are not shared with Art v 1. The general stronger immunogenicity of Art v 1 might be attributed to its abundance in the pollen but also to the higher stability of the immunologically relevant defensin domain during endolysosomal processing [18••]. In sunflower pollen, a 34-kDa protein termed Hel a 1 (no sequence available) was recognized by patients’ IgE from sunflower workers allergic to mugwort pollen [36]. Art v 1-specific antibodies confirmed the presence of a cross-reactive protein in sunflower pollen [37].

### IgE Cross-Reactivity of Pollen and Plant Food Defensins

Assessment of molecule-based IgE cross-reactivity between pollen and plant food defensins is currently only available for Art v 1 and defensins from celeriac and horse chestnut, sharing 63% and 57% sequence identity, respectively (Figs. 1 and 2) [34••]. Api g 7 is of high interest as it was tested positive in eight celeriac allergic patients with clinical symptoms. Notably, only three of these patients tested positive for celery extract, and no correlation between Api g 7 and the celeriac extract was observed. In contrast, a strong correlation was found for Api g 7 and Art v 1 reactivity. In ELISA cross-inhibition assays, Art v 1 was able to entirely abolish IgE binding to Api g 7 in three tested sera. On the other hand, Api g 7 only partially inhibited IgE binding to immobilized Art v 1 indicating that patients were primary sensitized to Art v 1 [34••].

Similar data was obtained for Art v 1 and horse chestnut seed defensin in a cohort of Aes h 1 sensitized subjects (Cosi et al., manuscript in preparation). While IgE reactivity of Aes h 1 did not correlate with horse chestnut seed extract, high correlation with Art v 1 was observed. IgE cross-inhibition showed that immobilized Aes h 1 could be efficiently inhibited by Art v 1, while this was only partially achieved when using Aes h 1 to block IgE binding to Art v 1. These data also suggest that Art v 1 acts as primary sensitizer. As seeds of horse chestnuts are used in traditional medicine, pharmaceutical and cosmetic products, and dietary supplements, this cross-reactivity is of high relevance for mugwort pollen-allergic patients.

In addition, two case reports on mango and sunflower seed allergy are noteworthy to mention as patients showed high reactivity to mugwort pollen extract and Art v 1. Inhibition experiments with either mango or sunflower seed extracts demonstrated inhibition of IgE binding to Art v 1 [38•, 39].

### Is There a Link Between Defensins and Severe Allergic Reactions, Including Anaphylaxis?

Identification of the underlying allergen in mugwort-associated food syndromes is of high relevance, as in many cases, severe food allergies including anaphylaxis are noted. IgE reactivity to Ara h 12/13 was predominately observed in patients with anaphylactic reactions to peanut [32•]. Among Chinese pollen-sensitized children with food anaphylaxis, 93.5% were sensitized to mugwort pollen with IgE levels significantly higher compared to ragweed and birch pollen [40]. Another study also demonstrated very high mugwort pollen sensitization frequencies in Chinese patients with anaphylaxis to fruits, vegetables, legumes, and spices [17]. Both studies however lack molecule-based analyses, and thus the potential role of defensins remains to be determined. A case report of a mugwort pollen-allergic patient with anaphylaxis to mango indicated a mango allergen at 13 kDa [41]. Analogous, a 12-kDa protein in garlic accounted for anaphylactic reactions to garlic consumption [42]. As molecule-based analysis using purified allergens was not performed in these studies, cross-reactivity with lipid transfer proteins cannot be fully ruled out. However, a later study revealed that an Art v 1 homolog in mango seems to be involved in the anaphylactic reaction [38•].

### Defensins as Missing Culprit Allergen in Mugwort Pollen-Associated Food Syndromes and Contact Allergies?

Up to now, four mugwort pollen food-associated syndromes are described, which are (i) celery–mugwort–spice syndrome, (ii) mugwort–chamomile association, (iii) mugwort–mustard syndrome, and (iv) mugwort–peach association. For the latter two syndromes, an involvement of lipid transfer proteins was suggested [43–47]. The potential involvement of defensins in the first two syndromes is discussed below.

The celery–mugwort–spice syndrome describes the clinical association between mugwort pollen and food allergy to vegetables and spices from the Apiaceae and Umbelliferae plant family. It includes adverse reactions to celery, carrot, parsley, fennel seeds, caraway seeds, coriander seeds, aniseeds, and later also includes paprika, pepper, onion, and garlic. However, the underlying allergen molecules could not unambiguously be identified [48–51]. Celery-allergic patients with a mugwort pollen allergy frequently display stronger IgE reactivity to heated celery extract [52–54]. This contrasted the finding that celery-birch patients display low or no reactivity to heated celery due to the low heat stability of the involved Api g 1. These findings strongly suggest the involvement of a highly stable cross-reactive allergen, and the recently identified Api g 7 from celeriac might fulfill these criteria. In line with the thermostability of defensins, Api g 7 demonstrates a melting point of 100 °C [34••]. Even though the current evidence is supported by a limited number of patients, Api g 7 might be the missing link in the celery–mugwort syndrome due to its structural similarity to Art v 1 and demonstrated IgE cross-reactivity.

Among 15 patients with allergic reactions upon consumption of chamomile tea, 12 were linked with mugwort pollen allergy describing the mugwort–chamomile association. On the other hand, among 24 mugwort allergic patients, 86% were skin prick test positive, and 62% showed oral allergy symptoms upon testing with chamomile. A later case study showed an association between anaphylaxis to chamomile tea and Art v 1 sensitization [55].

In addition, cases of allergic reactions including anaphylaxis to artisanal honey were reported in patients sensitized to mugwort pollen, suggesting cross-reactive components in honey but more likely a contamination of the honey with *Artemisia* pollen [17]. As Art v 1, which was not tested in this case, is a highly stable molecule, it might also be involved in food-related symptoms. However, the involvement of lipid transfer proteins cannot be fully ruled out, even though Pru p 3 was tested negative.

Based on our unpublished data on IgE cross-reactivity of Art v 1 and Aes h 1, we extend the palette of mugwort-associated allergies to horse chestnut belonging to the Sapindaceae family. Mango belongs to the Anacardiaceae family and should therefore not be included in the celery–mugwort–spice syndrome as it might present an independent pollen food syndrome [38•]. IgE cross-reactivity of peanut and soybean defensins with pollen DPLP was so far not tested. The sequence identity with Art v 1 is rather low (< 32%) and thus potentially not sufficient for considerable cross-reactivity. Despite a lower allergenic relevance of Amb a 4, it might also be worth testing food-related IgE cross-reactivity especially in patients highly exposed to ragweed pollen.

### Are Defensins Underrepresented But Highly Relevant Food Allergens?

There is increasing evidence that defensins could be involved in mugwort pollen-related food syndromes. While large protein amounts of natural Art v 1 can be obtained, the discovery and purification of food defensins seem more challenging. While the expression of defensins might vary in response to the plant’s stress levels and pathogenic exposure, it also seems likely that typically used aqueous extraction protocols are insufficient for accurate allergy diagnosis [14]. Particularly extraction from seeds and tuber might be hampered by matrix effects and the presence of glycans, polyphenols, or high lipid content [32•, 34••]. In addition, the use of standard gel electrophoresis under reducing conditions typically disrupts conformational epitopes of defensins and thus hampers recognition by patients’ IgE. This might explain why only few allergenic defensins from food were identified and characterized. However, once identified, they can be produced as recombinant proteins, characterized and used for molecule-based allergy diagnosis. The role of defensins in pollen food syndromes might therefore be underrated as typically lipid transfer proteins are firstly considered when severe symptoms and stable allergens are observed.

### Diagnostic Options

Extract-based diagnosis of pollen allergy involving DPLP is limited, as apart from Art v 1; defensins do not constitute major components of the respective extracts. In addition, patients allergic to weeds are typically poly-sensitized, and only molecule-based diagnosis allows discrimination from Amb a 1-related weed pollen allergy [10]. In the clinic, Art v 1 molecule-based diagnosis is available as purified natural (IgE singleplex and multiplex ImmunoCAP) or recombinant (IgE multiplex ALEX) allergen. Purified recombinant Amb a 4 is available in the ALEX multiplex format.

With respect to defensins in plant foods, those allergens seem underrepresented in aqueous extracts typically used for diagnosis [34••]. Interestingly, prick-to-prick tests using fresh fruits and vegetables usually allow monitoring a reaction; however, this may not always be linked to defensins. On the other hand, food allergies with unclear culprit allergens are often associated with Art v 1 sensitization [38•, 39]. There is an urgent need for identification and recombinant production of additional allergenic defensins in edibles, especially from sources triggering severe food allergies. At present, Art v 1 and Amb a 4 could be used as a surrogate marker for food allergies with unclear elicitor.

### Therapeutic Options

Regarding allergen-specific immunotherapy, predominately *Artemisia* spp. extracts represent a commonly used therapeutic agent for the treatment of defensin-related weed pollen allergy. Amb a 4 — in contrast to Art v 1 — is a minor allergen with limited quantity in ragweed extract that frequently cross-reacts with Art v 1. However, there seems to be a small sub-group of patients with IgE to unique Amb a 4 epitopes as they seem primarily sensitized to ragweed defensin. For those patients, immunotherapy with Amb a 4 containing ragweed extract would be favorable [18••]. For potential novel treatment options, hypoallergenic variants of Art v 1 were generated by targeting the disulfide bond-stabilized structure [23, 56]. In a murine model, virus-like nanoparticles with shielded Art v 1 were hypoallergenic and successful in preventive treatment [57]. A study also showed that the adjuvant is relevant for a successful outcome in allergen-specific immunotherapy in mice [58]. Food allergies involving defensins have not been investigated in oral immunotherapy protocols. Avoidance of the culprit food and use of adrenaline for severe cases are typically recommended.

## Conclusion

For decades, mugwort pollen-associated food symptoms were clinically recognized, yet the causative molecules were not elucidated. Recent studies suggest that defensins could be involved in those syndromes leading to severe allergic reactions. It seems reasonable that Art v 1 and highly homologous allergens in more than 350 *Artemisia* species account for the primary sensitization due to their high abundance. Climatic and pathogen stress might increase the expression of defensins, potentially leading to more sensitizations and allergic reactions.

Within the Asteraceae family, IgE cross-reactivity of mugwort with ragweed, feverfew, sunflower, and chamomile was observed at varying degrees. So far, Art v 1-related food and contact allergies have been reported for celeriac, horse chestnut seed, mango, and sunflower seed allergic patients. In contrast to other pollen food syndromes, cross-reactivity with Art v 1 is not limited to specific plant families but rather to the highly conserved 3-dimensional structure. To distinguish from other mugwort-associated pollen food syndromes, we propose the novel term “defensin-related food allergy.” In light of the severe allergic reactions, the identification of further allergenic defensins is urgently needed. This will allow molecule-based allergy diagnosis with recombinant proteins to provide a better understanding of defensin-related food allergies and awareness for enhanced patients’ recommendation.
